# Sjögren: unique surname, two men, four syndromes and one disease

**DOI:** 10.1055/s-0044-1786022

**Published:** 2024-04-23

**Authors:** José Vitor Alécio Rodrigues, Fábio Antônio Serra de Lima, Daniel Pereira Maurício de Barros, Gustavo Leite Franklin, Adriana Meira Tiburtino Nepomuceno, Alessandra de Sousa Braz, Hélio A. G. Teive, Alex T. Meira

**Affiliations:** 1 Universidade Federal da Paraíba, Centro de Ciências Médicas, João Pessoa PB, Brazil.; 2 Pontifícia Universidade Católica do Paraná, Departamento de Medicina Interna, Serviço de Neurologia, Curitiba PR, Brazil.; 3 Universidade Federal da Paraíba, Departamento de Medicina Interna, Serviço de Reumatologia, João Pessoa PB, Brazil.; 4 Universidade Federal do Paraná, Serviço de Neurologia, Curitiba PR, Brazil.; 5 Universidade Federal da Paraíba, Departamento de Medicina Interna, Serviço de Neurologia, João Pessoa PB, Brazil.

**Keywords:** Sjogren's Syndrome, Retinal Pigment Epithelium, Sjogren-Larsson Syndrome, Neuronal Ceroid-Lipofuscinoses, Spinocerebellar Degenerations, Síndrome de Sjogren, Epitélio Pigmentado da Retina, Síndrome de Sjogren-Larsson, Lipofuscinoses Ceroides Neuronais, Degenerações Espinocerebelares

## Abstract

Henrik and Torsten Sjögren (/ˈʃoʊɡrən/ or SHOH–grən) were two Swedish physicians living in the same period, but completely unrelated, except for their notable contributions to Medicine. The first one described keratoconjunctivitis sicca, afterward called Sjögren's syndrome, and a fishing net aspect retinal pigmentation affecting visual acuity, nowadays known as Sjögren reticular dystrophy. The last one contributed to the understanding of Spielmeyer-Sjögren disease, Marinesco-Sjögren, and Sjögren-Larsson syndromes, all related to genetic disorders and neurological symptoms. In this paper, we aim to describe each disorder, in order to avoid any misunderstanding in diagnosis and for historical record.

## Resumo

Henrik e Torsten Sjögren (/ˈʃoʊɡrən/ ou SHOH-grən) foram dois médicos suecos que viveram na mesma época, mas não tinham nenhuma relação entre si, exceto por suas notáveis contribuições à medicina. O primeiro descreveu a ceratoconjuntivite sicca, posteriormente chamada de síndrome de Sjögren, e uma pigmentação da retina com aspecto de rede de pesca que afeta a acuidade visual, hoje conhecida como distrofia reticular de Sjögren. O último contribuiu para a compreensão da doença de Spielmeyer-Sjögren, das síndromes de Marinesco-Sjögren e Sjögren-Larsson, todas relacionadas a distúrbios genéticos e sintomas neurológicos. Neste artigo, pretendemos descrever cada desordem, a fim de evitar qualquer mal-entendido no diagnóstico e para registro histórico.

## INTRODUCTION

**Figure 1:**
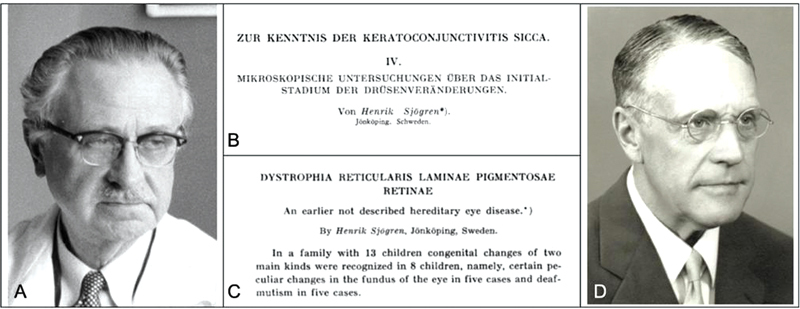
( **A** ) Portrait of Henrik Samuel Konrad Sjögren (1899–1986; by Per Weimersson, 1958). ( **B** ) Chapter IV of the H. Sjögren's doctoral thesis, “Zur Kenntnis der keratoconjunctivitis sicca”, published in 1933, originally in German. The content widespread worldwide only in 1943, after the English translation by John Bruce Hamilton under the title “A New Conception of Keratoconjunctivitis Sicca”. ( **C** ) The article by H. Sjögren in 1950 about the Sjögren reticular dystrophy. ( **D** ) Portrait of Karl Gustaf Torsten Sjögren (1896–1974).

The surname Sjögren is rare outside Sweden and Finland. Two physicians with this surname became famous for describing four syndromes and one disease. The Swedish Henrik Samuel Konrad Sjögren (1899–1986; Figure 1A ) graduated in Medicine in 1918 from the Karolinska Institute and was passionate about ophthalmology and published his case series of keratoconjunctivitis sicca and its association to dry mouth and polyarthritis ( Figure 1B ). 1 Another Swedish Karl Gustaf Torsten Sjögren (1896–1974; Figure 1D ) graduated in Medicine in Stockholm in 1925 and became a Doctor of Medicine in 1931 from Lund University and focused his career on psychiatry and genetics. He established a psychiatric unit in Gothenburg and described three conditions: Sjögren-Larsson syndrome, Marinesco-Sjögren syndrome, and Spielmeyer-Sjögren disease. 2 This study aims to approach the five disorders to which the two physicians contributed, analyzing the historical aspects of each one and their contribution.

This is a narrative review based on searching articles in main databases (PubMed, LILACS, Research Gate, ScienceDirect, Web of Science, Embase, Google Scholar, and SciELO) using “Sjogren”, “Sjögren, “Sjogren Syndrome”, “Henrik Samuel Konrad Sjögren”, and “Karl Gustaf Torsten Sjögren” as keywords. No filter was applied regarding the year of the published article, type of article, and language. However, English articles were preferred. Some textbooks and the English version of the Royal Swedish Academy of Sciences( https://www.kva.se/en/ ) were consulted. We avoid missing syndromes or diseases involving the surname Sjögren actively searching on the Online Mendelian Inheritance in Man - OMIM (omim.org) and the Orphanet Rare Diseases Ontology - ORDO ( https://www.orpha.net ).

## SICCA SYNDROME (SJÖGREN'S SYNDROME)

Henrik Sjögren was the first to describe a group of women and correlate the triad of dry eyes, dry mouth, and polyarthritis. Hadden and Hutchinson reported the first case of dry eyes and dry mouth in 1871. Johann von Mikulicz–Radecki described the enlargement of the parotid, submandibular, and lacrimal glands, which became known as Mikulicz disease, a subset of Sjögren's syndrome. Henri Gougerot reported the classical triad for the first time in 1925 and went beyond, reporting systemic involvement of the syndrome as more glandules could be affected. Some authors later called the condition Gougerot or Gougerot-Sjögren syndrome. 3 4

In his doctoral thesis, Henrik Sjögren described 19 female cases of dry eyes and dry mouth and proposed some treatments, in 1935. The most important finding of his thesis was that the syndrome was not restricted to the eye but associated with the salivary glands which presented diminished secretion. This finding expanded the understanding of the syndrome's systemic implications. 1 3

Despite previous reports on keratoconjunctivitis sicca, it was Henrik Sjögren's contribution in correlating it with xerostomia and polyarthritis that was so remarkable that he superseded his antecedents in being coined the name of the syndrome.

## SJÖGREN RETICULAR DYSTROPHY

A rare reticular dystrophy characterized by hyperpigmentation of the retinal pigment epithelium, gradually from the posterior pole to the peripheral retina in a fishing net aspect with unaffected choriocapillaris. 5

In 1950, H. Sjögren was the first to describe a family of 8 children, in which 5 had peculiar changes in the fundoscopy, 5 had congenital deafmutism, 3 had an atrophied pigmented layer of the iris and 2 presented spherophakia. On examination with red-free and green light, the network was indetectable, while was hardly discernible in the stronger light of a mercury lamp, suggesting it was located very deeply. During follow-up, some patients had an increase in network diameter, suggesting a progressive component in the syndrome ( Figure 1C ). 6

Currently, the diagnosis includes bilateral and symmetric onset, centrifugal progression; progressive reduction of reticular pattern and normal retinographic findings. It is important to point out that this condition is not related to the sicca syndrome. 7

## SPIELMEYER-SJÖGREN DISEASE (BATTEN DISEASE)

Spielmeyer-Sjögren disease is a neuronal ceroid lipofuscinosis, a lysosomal storage disease that affects the retina associated with progressive brain degeneration. The clinical features are functional visual deficiency (retinitis pigmentosa) with onset between 5 and 10 years, which evolves into amaurosis (retinal degeneration) and then cognitive impairment. 8

Possibly the first report of Spielmeyer-Sjögren disease was made by Otto Christian Stengel in 1826, who described a juvenile-onset disease that evolved with blindness and dementia in four children. 9 In 1903, Frederick Batten described the neuropathology of the neuronal storage disease leading to retinal and cerebral degeneration with macular changes. 10 All these cases were included among the group of diseases called amaurotic family idiocy, a group of diseases distinguished by the accrual of lipids in visceral organs and the nervous system, accompanied by blindness or visual impairment, as well as cognitive decline. Only in 1931, Torsten Sjögren carried out extensive clinical and genetic studies demonstrating that these disorders had a monohybrid recessive inheritance mode, distinguishing genetically from the infantile amaurotic idiocy. Therefore, the juvenile amaurotic idiocy became more broadly known as Batten disease (or Spielmeyer-Vogt-Sjögren-Batten disease). 11

## MARINESCO-SJÖGREN SYNDROME

Marinesco-Sjögren is a rare genetic disorder affecting multiple body systems, resulting in balance problems, cataracts from birth, muscle weakness, learning and growth difficulties, leading to intellectual disability, and short stature. 12

The syndrome was first reported by Ernst Emil Moravscik in 1904, who studied three siblings with cataracts, eye problems, and neurological symptoms. They also had low height, spinal curvature, and delayed puberty. 13

In 1931, Marinesco et al. added other findings, such as hypogonadism, chest deformity, and flat feet. 14 In 1947 and 1949/1950, Torsten Sjögren advanced the understanding of the syndrome phenotype. He found three families (16 individuals) with patients who inherited the syndrome recessively and had frequent consanguineous marriages. 15 16 We recommend the inclusion of Moravscik's name in Marinesco-Sjögren syndrome, due to his pioneering and substantial contributions.

## SJÖGREN-LARSSON SYNDROME

Sjögren-Larsson syndrome is an autosomal recessive neurocutaneous disorder caused by mutations in the ALDH3A2 gene that affect the fatty aldehyde dehydrogenase synthesis, resulting in overproduction of long-chain aliphatic aldehydes and alcohols. The classical triad is spastic diplegia or tetraplegia, mental retardation, and congenital ichthyosis. 17

T. Sjögren was the first to describe Sjögren-Larsson syndrome in 1956, in the northeast of Sweden. The study documented a cohort of 25 individuals from 10 unrelated families exhibiting the classical triad. 18 Sjögren and Larsson described 28 patients in their long monograph, recognizing the autosomal recessive inheritance of the syndrome. In 1974, Sjögren-Larsson syndrome was recognized outside Sweden, well distributed in different localities and distinct ethnic groups. 19 Later in 1988, the disorder was found to be the first inherited disorder in man correlated with an isolated abnormality in fatty alcohol metabolism. 20

In conclusion, Henrik and Torsten Sjögren were not the first to describe most of the disorders that bear their surnames. However, their contributions were crucial in gathering, describing, and analyzing the key elements that make up the conditions, providing the basis for more detailed studies that complemented their findings. This study has brought together 5 different conditions with different clinical features, but with important genetic and, in 4 of them, neurological bases described by 2 different authors. These disorders are now better described from etiology to treatment, as well as much of the history of studies that made up this knowledge. Finally, due to his pioneering and substantial contributions, we recommend the inclusion of Moravscik's name in Marinesco-Sjögren syndrome.
